# Effect of hyperglycaemia in combination with moxifloxacin on cardiac repolarization in male and female patients with type I diabetes

**DOI:** 10.1007/s00392-022-02037-8

**Published:** 2022-05-21

**Authors:** Jorg Taubel, Dominic Pimenta, Samuel Thomas Cole, Claus Graff, Jørgen K. Kanters, A. John Camm

**Affiliations:** 1 St George’s University of London, London, UK; 2 Richmond Pharmacology Ltd, London, UK; 3 Richmond Research Institute, St George’s University, London, UK; 4 Department of Health Science and Technology, Aalborg University, Aalborg, Denmark; 5 Department of Biomedical Sciences, University of Copenhagen, Copenhagen, Denmark

**Keywords:** Type 1 diabetes mellitus, QTc, Sudden cardiac death, Hyperglycaemia, Sex differences, Potassium

## Abstract

**Background:**

Patients with Type 1 diabetes mellitus have been shown to be at a two to ten-fold higher risk of sudden cardiac death (SCD) (Svane et al., Curr Cardiol 2020; 22:112) than the general population, but the underlying mechanism is unclear. Hyperglycaemia is a recognised cause of QTc prolongation; a state patients with type 1 diabetes are more prone to, potentially increasing their risk of ventricular arrhythmia. Understanding the QTc prolongation effect of both hyperglycaemia and the concomitant additive risk of commonly prescribed QTc-prolonging drugs such as Moxifloxacin may help to elucidate the mechanism of sudden cardiac death in this cohort. This single-blinded, placebo-controlled study investigated the extent to which hyperglycaemia prolongs the QTc in controlled conditions, and the potential additive risk of QTc-prolonging medications.

**Methods:**

21 patients with type 1 diabetes mellitus were enrolled to a placebo-controlled crossover study at a single clinical trials unit. Patients underwent thorough QTc assessment throughout the study. A ‘hyperglycaemic clamp’ of oral and intravenous glucose was administered with a target blood glucose of > 25 mM and maintained for 2 h on day 1 and day 3, alongside placebo on day 1 and moxifloxacin on day 3. Day 2 served as a control day between the two active treatment days.

Thorough QTc assessment was conducted at matched time points over 3 days, and regular blood sampling was undertaken at matched time intervals for glucose levels and moxifloxacin exposure.

**Results:**

Concentration-effect modelling showed that acute hyperglycaemia prolonged the QTc interval in female and male volunteers with type 1 diabetes by a peak mean increase of 13 ms at 2 h. Peak mean QTc intervals after the administration of intravenous Moxifloxacin during the hyperglycaemic state were increased by a further 9 ms at 2 h, to 22 ms across the entire study population. Regression analysis suggested this additional increase was additive, not exponential.

Hyperglycaemia was associated with a significantly greater mean QTc-prolonging effect in females, but the mean peak increase with the addition of moxifloxacin was the same for males and females. This apparent sex difference was likely due to the exclusive use of basal insulin in the male patients, which provided a low level of exogenous insulin during the study assessments thereby mitigating the effects of hyperglycaemia on QTc. This effect was partially overcome by Moxifloxacin administration, suggesting both hyperglycaemia and moxifloxacin prolong QTc by different mechanisms, based on subinterval analysis.

**Conclusions:**

Hyperglycaemia was found to be a significant cause of QTc prolongation and the additional effect of a QTc-prolonging positive control (moxifloxacin) was found to be additive. Given the high risk of sudden cardiac death in type 1 diabetes mellitus, extra caution should be exercised when prescribing any medication in this cohort for QTc effects, and further research needs to be undertaken to elucidate the exact mechanism underlying this finding and explore the potential prescribing risk in diabetes.

**Trial Registration:**

NCT number: NCT01984827.

**Graphical abstract:**

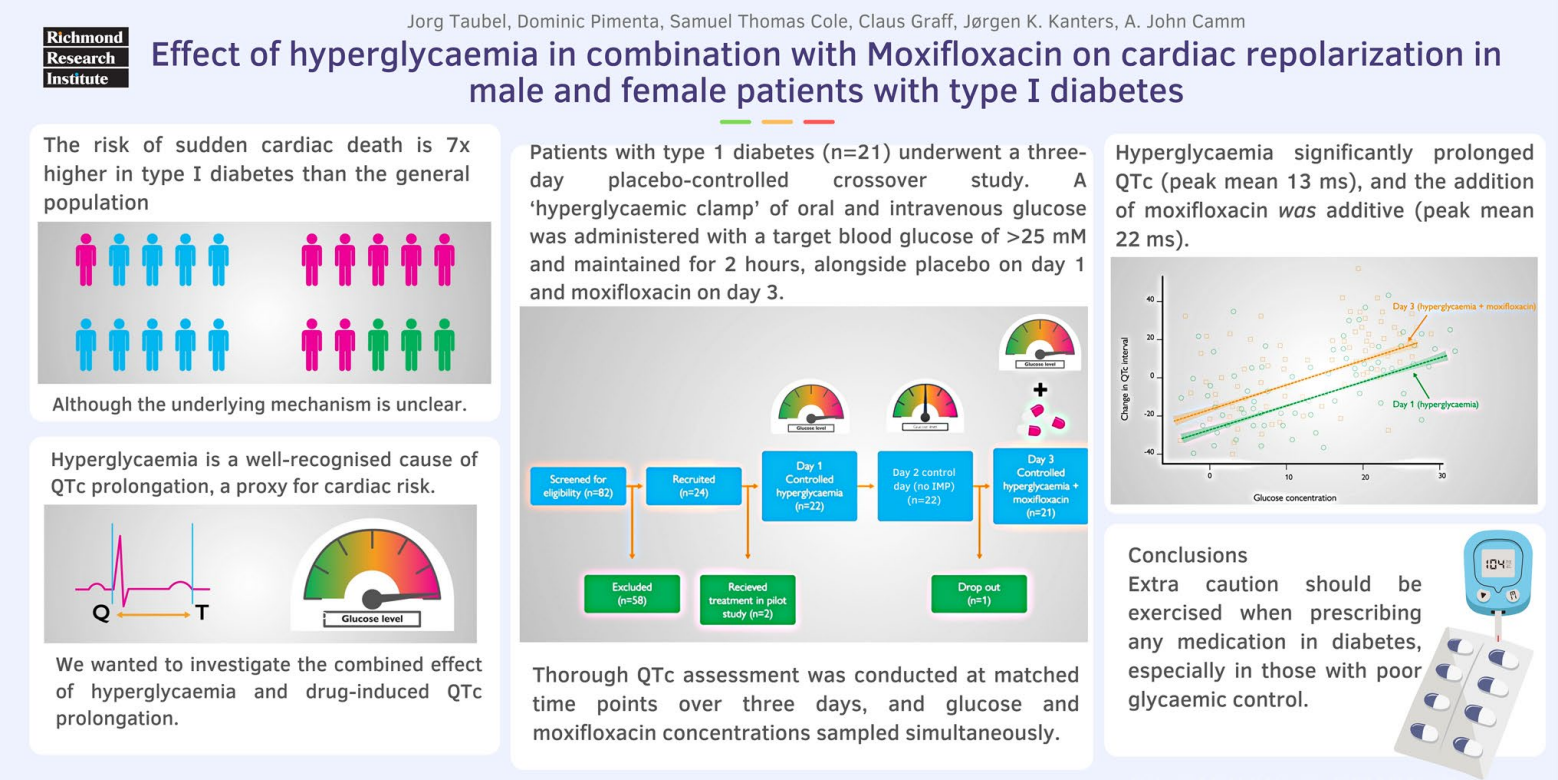

**Supplementary Information:**

The online version contains supplementary material available at 10.1007/s00392-022-02037-8.

## Introduction

Diabetes mellitus (DM) is a common metabolic disorder characterised by high blood glucose. It is caused by a lack of pancreatic beta-cell insulin production in type 1 DM, or a reduced sensitivity of tissues to insulin in type 2 DM. Patients with type 1 diabetes mellitus have a seven-fold higher risk of sudden cardiac death than age-matched non-diabetic controls [37], although the underlying mechanism for this is unclear. Sudden cardiac death is closely linked with cardiac arrhythmia, particularly Torsades de Points (TdP) and prolongation of the QT interval is a key risk factor.

### QTc prolongation in type 1 diabetes mellitus (DM)

**Fig. 1:**
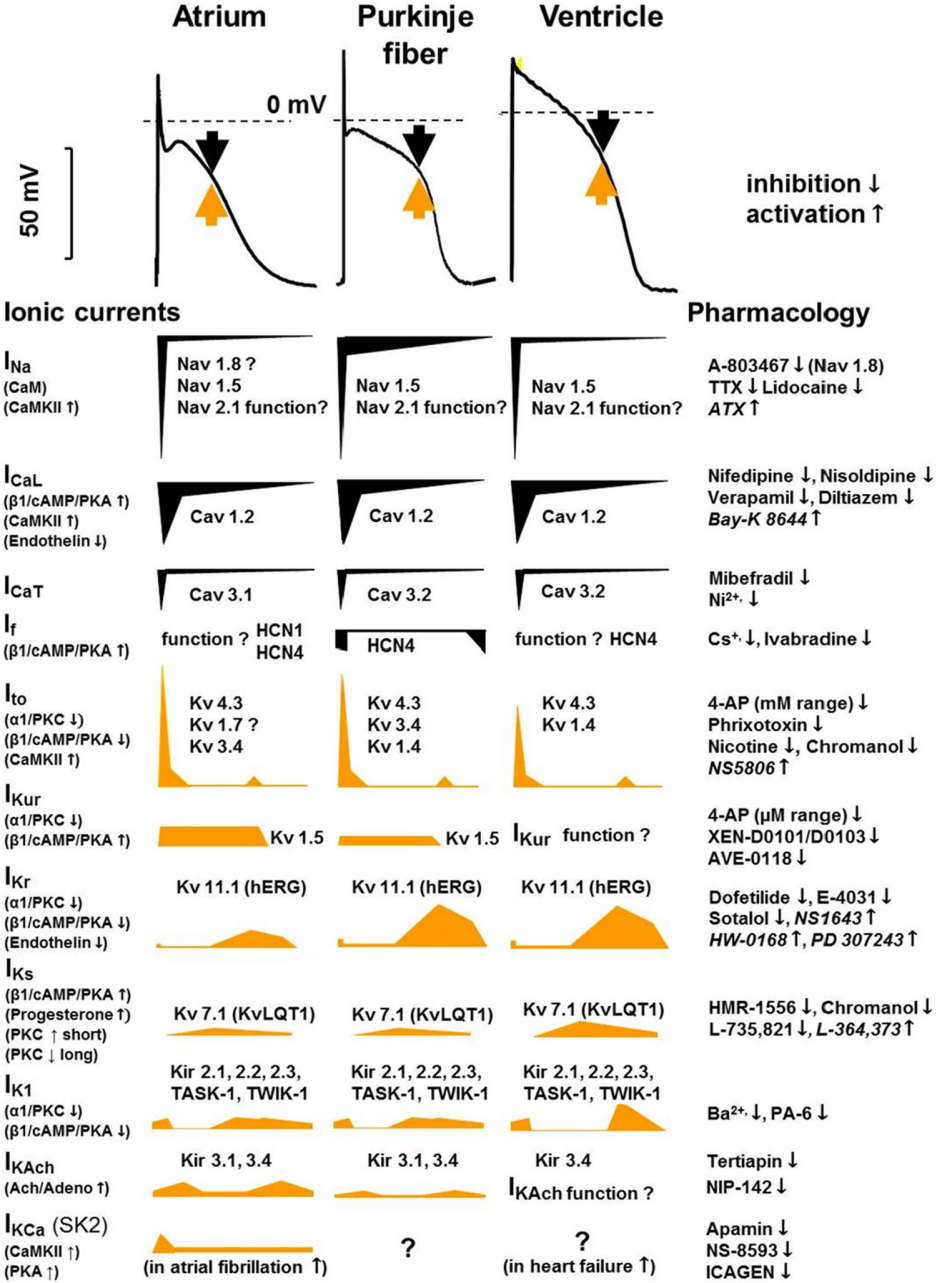
Reprinted with permission [42]. “Tissue-specific (human) cardiac atrial, Purkinje fiber, and ventricular action potentials and the underlying ionic currents in different action potential phases, indicating their pharmacology and modulation. Black arrows indicate inward and yellow arrows indicate outward current. The contributions of different currents to the action potentials are indicated below, with a time course adjusted to the action potential. *CaM* calmodulin *CaMKII*, *Ca2* + -calmodulin kinase II, *hERG* human ether-à-go-go-related gene, *IK1* inward rectifier potassium current, *IK,Ach* acetylcholine-activated potassium current, *INa* sodium current, *ICaL* L-type calcium current, *ICaT* T-type calcium current, *If* funny/pacemaker current, *Ito* transient outward current, *IKCa* calcium-activated potassium current, *IKr, IKs* and *IKur* rapid, slow, and ultrarapid components of delayed rectifier potassium current, *Kir* inward rectifier potassium channel, *KV* voltage-gated potassium channel, *NaV* voltage-gated sodium channel, *TASK* Tandem of pore domains in a weak inward rectifying potassium channel (*TWIK*)-related acid-sensitive potassium channel, *TTX* tetrodotoxin.”

Cardiac myocytes depolarize and repolarize through the movement of positively charged ions (Na + , K + , Ca2 +) via a variety of ion channels within the cell membrane. (Fig. 1 – Action Potential and associated ion channels) [42]. Defects in ion channel function alter repolarization, reflected in the 12-lead surface ECG as a prolonged QT interval.

Experimental evidence in animal models and clinical studies of type 1 and type 2 DM has demonstrated multiple cellular, hormonal, inflammatory, neuropathic and physiological processes (Fig. 1 – Supplementary material) that influence the QTc via ion channel modulation, particularly the K + ion channels IK_r_, IK_s_ and Ito.

In type I diabetes, multiple studies have demonstrated an association with hyperglycaemia and QTc prolongation. Continuous glucose monitoring paired with continuous ECG recording in T1DM patients demonstrated QTc prolongation occurring with hyperglycaemia at various points in the day [4], however, other similar real-world studies have shown no such effect [1].

Possible factors explaining such discrepancies in the literature include the study technique, the study setting (clinic versus real-world) and the QTc measurement methodology used, for example Holter monitor versus continuous in-house telemetry, the latter being far more accurate. Additionally, various cut-off definitions of hyperglycaemia are utilised in controlled experiments, ranging from 15 to 25 mM, and this makes the literature highly heterogenous.

In adolescents, QTc prolongation was positively correlated with HBA1c [35] suggesting this is an early onset effect tied to hyperglycaemia exposure. Similarly, early-stage type II diabetes mellitus patients showed autonomic neuropathy associated with QTc prolongation [12], and in a population-based study blood glucose level was an independent significant risk factor for severe QTc prolongation (> 500 ms) [26]. Chronic swings in high to low glucose levels, so-called ‘glycaemic variability’ [36], also appears to be a significant risk factor for QTc prolongation, with post-prandial glucose levels a better predictor of QTc prolongation than fasting glucose alone.

Fluroquinolones are associated with varying degrees of QTc prolongation [39], with several of the class withdrawn from market due to severe prolongation. This effect has been mostly hypothesised to centre on the ion channel IK_r_ [6]. Moxifloxacin is often used a positive control in thorough QTc studies to evaluate assay sensitivity [25]. No clinical studies have looked at the additive effect of known QTc-prolonging medication and hyperglycaemia under controlled conditions, despite large groups of the population routinely exposed to both. This is underlined by the fact that the effect of fluroquinolones on the QTc in real-life studies appears to behave differently between diabetics and non-diabetics [33].

This study aimed to explore the combined effects of hyperglycaemia and the administration of a QT-prolonging drug, and to gain insight into potential mechanisms by using the state-of-the-art subinterval measurements. Additionally, the study aimed to explore whether sex differences play a role in the extent of QT prolongation in Type 1 DM, as it has previously been observed that women can experience a greater extent of prolongation in response to QT-prolonging drugs and, therefore, may be at greater risk of developing TdP than men.

Our primary objective, therefore, was to evaluate the effect of hyperglycaemia (using a controlled ‘clamp’ technique) and a QTc-prolonging medication (moxifloxacin) on the QTc interval in type 1 DM. To explore the underlying mechanisms, our exploratory objectives included the examination of sex differences. We also investigated the influence of the patients’ exogenous insulin regimen on QTc and its subintervals.

## Methods

### Study design

This was a single-blind, single‐centre, randomised, placebo‐controlled study conducted at Richmond Pharmacology, London, UK, which assessed the QTc-prolonging effects of a combined oral and intravenous hyperglycaemic glucose clamp alone and in combination with moxifloxacin when administered to patients with type 1 DM (NCT01984827).

**Fig. 2:**
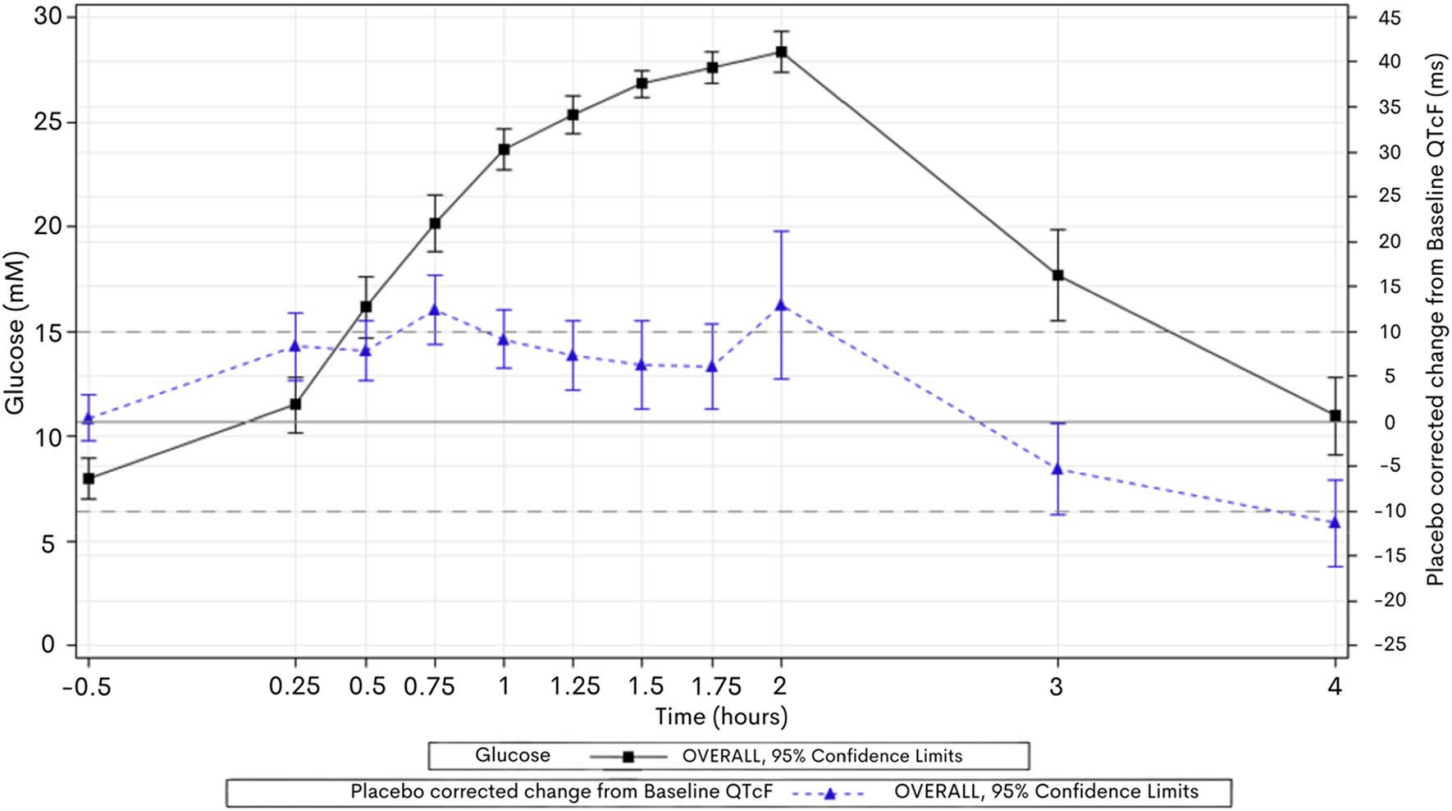
Mean ∆∆QTcF over time vs Mean Glucose concentration over time, on day 1 (glucose administration only). Volunteers were administered hyperglycaemic clamp and QTcF compared to placebo on day 2. Peak mean placebo corrected increase in QTcF was 13 ms at peak glucose concentration (2 h)

The study schedule is shown in Fig. 2—Supplementary material.

Patients with type 1 diabetes mellitus underwent an oral and intravenous hyperglycaemic clamp plus a placebo infusion on day 1, received a placebo infusion only on day 2, and underwent a second oral and intravenous hyperglycaemic clamp plus 300 mg intravenous moxifloxacin (over 45 min) on day 3. On days 1 and 3, glucose was administered orally first, followed by an intravenous maintenance infusion with the aim of gradually raising glucose concentration to a target range of 25 mM over the initial 60 min. The 25 mM glucose level was then maintained for a further 60 min, titrating IV glucose as guided by glucose readings. Moxifloxacin or a matching placebo was given at the 1 h timepoint, by intravenous infusion over 45 min. At the end of 2 h, a small bolus of Novorapid insulin was given and an Actrapid variable rate intravenous insulin infusion (VRIII) and potassium replacement fluid (10 mmol/h) was commenced and continued until the blood glucose levels decreased to 8 mM.

Volunteers were instructed to maintain any existing long-acting insulin regimen over the course of the trial and volunteers using a bolus or subcutaneous pump regimen did not take their short-acting insulin on the morning of days 1 and 3 but used their normal regimen on day 2. Those using an insulin pump had their pump disconnected prior to commencing the clamp, and reconnected when glucose levels returned to normal range following the VRIII. Patients on a basal-bolus regimen continued their basal long-acting insulin throughout the study, and therefore, had low levels of background insulin during the clamp experiment.

Volunteers were screened within 20 days prior to entering the study. Each volunteer received verbal and written information and signed an Informed Consent Form prior to any screening procedures taking place. Volunteers were admitted to the unit on day − 1, dosed on days 1–3 and discharged on day 4. All volunteers returned for a follow-up visit 7–14 days from day 1.

The study protocol was reviewed and approved by a National Health Service Research Ethics Committee. The study was conducted according to the ethical principles enshrined in UK law, the Declaration of Helsinki, and Good Clinical Practice guidelines. Participants were given written and verbal information before signing an informed consent and advised that they could leave the study at any point if they so wished.

### Study participants

**Fig. 3:**
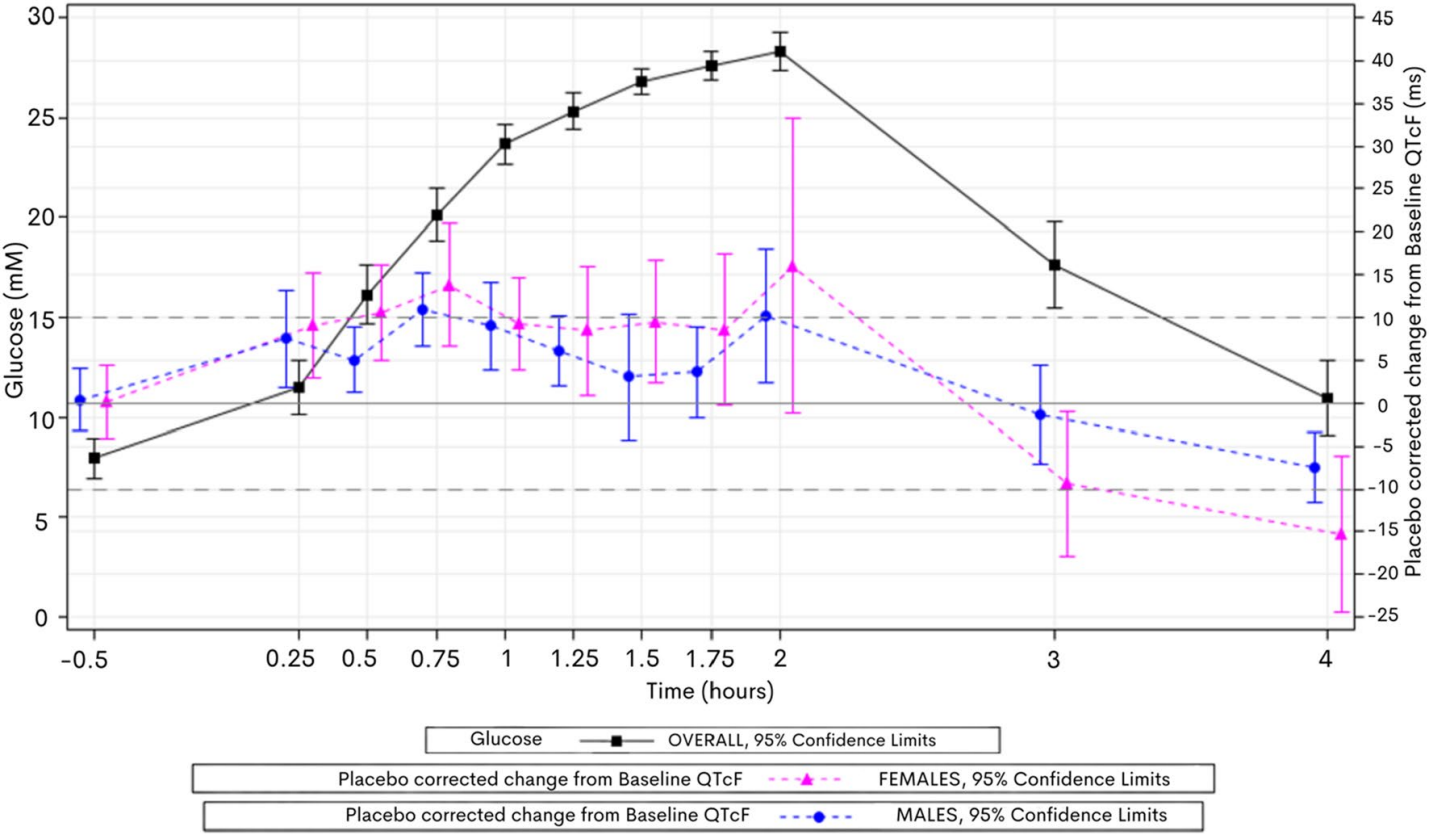
Mean ∆∆QTcF over time vs Mean Glucose over time on day 1—by Gender. Peak female placebo corrected QTcF increase at 2 h was 16 ms compared to males (10 ms)

Participants (12 females, 10 males) with a confirmed diagnosis of type 1 diabetes mellitus, who were otherwise well were included in this study. One individual dropped out on day 2 and her data were not included in any analysis sets. The remaining 11 females and 10 males completed all assessments; demographics are shown in Table 1—Supplementary material and the consort chart in Fig. 3—Supplementary material.

Participants were judged to be otherwise healthy from a physical examination, routine laboratory assessments, and screening ECG. Patients had to have normal sinus rhythm, without bundle branch block (QRS < 120) or pre-existing QTc prolongation (QTcF < 450 ms), to be included in the trial. Exclusion criteria included any pathology or abnormality with possible influence on the ECG and use of any concomitant medications with QT-prolonging effects, poorly controlled diabetes or diabetic complications. There were no washout intervals between study days. All patients’ glucose levels were monitored continuously using a continuous glucose monitoring system (CGMS) (Dexcom) to ensure the safety of participants in addition to regular measurements using blood from a cannula.

### Cardiac assessments and ECG analysis

Intensive cardiac assessments and analysis of changes in the QTcF interval relative to glucose concentrations were performed on days 1–3 to evaluate the effect of hyperglycaemia and moxifloxacin individually and in combination on the QTc interval. All ECG recordings were obtained in triplicate, performed at 1-min intervals over three minutes for each time point to confirm accuracy and precision of measurements. Electrocardiograms used for this analysis required adjudication by qualified cardiologists in a blinded fashion, in line with the principles set out in the International Conference on Harmonisation (ICH) E14 guideline.

ECG recordings were collected at 2-, 1-, and 0.5-h pre-dose and at 0.25, 0.5, 0.75, 1, 1.25, 1.5, 1.75, 2, 3, 4, 5, 6, 7, 8, 9, 10, and 12 h post-dose. All ECGs were recorded when volunteers had been resting and were awake in a supine position for at least 10 min, avoiding postural changes.

The 10 s 12-lead ECGs were recorded with the subject in supine position after a 10-min resting period and prior to the PK sampling. The 12-lead ECGs were recorded using an electrocardiograph (MAC-1200; General Electric Healthcare, Milwaukee, USA) and 10 disposable electrodes placed in the standard anatomical position. The ECG data were then processed by the Department of Health Science and Technology, Faculty of Medicine, University of Aalborg (Denmark) using the commercially available GE Healthcare Marquette 12SL ECG analysis program and the US Food and Drug Administration 510(k)-cleared GE research package QT Guard Plus (GE Healthcare, Wauwatosa, WI, USA), which uses validated algorithms for measurement of ECG parameters. The software uses all simultaneous 12 leads to construct a representative median beat from non-ectopic PQRST complexes and measures intervals from the earliest onset in any lead to the latest offset in any lead. Parameters that were automatically assessed in the ECG were: RR, QT, PR, QRS, J-Tpeak (JTp), Tpeak-Tend (TpTe) intervals. The QT interval was corrected for heart rate using the Fridericia method ($\mathrm{QTcF}=\mathrm{QT}/\sqrt[3]{\mathrm{RR}}$), in line with ICH E14 guidance where HR changes are small. The JTp interval was corrected for heart rate using the Johannesen formula $\left(\mathrm{JTpc}=\frac{\mathrm{J}-\mathrm{ Tpeak}}{{\mathrm{RR}}^{0.58}}\right)$ (JTp_c_J) [19]. A total of 5820 ECGs were used to calculate 1171 triplicate means after adjudication. The average of the three pre-clamp values was taken for use as a baseline.

### Pharmacokinetics

Venous blood samples were collected at 0.5 h pre-dose and at 0.25, 0.5, 0.75, 1, 1.25, 1.5, 1.75, 2, 3, 4, 5, 6, 7, 8, 9, 10, and 12 h post-dose. Serum glucose and potassium were measured using a blood gas analyser (ABL90 FlexPlus, Radiometer Medical). Sodium, and calcium concentrations and C-peptide were analysed using validated methods (SST sample clotted for 30 min, centrifuged at 2000 g at 18–25 ºC and the serum separated into a transfer vial) and then transferred to an off-site laboratory (The Doctors’ Laboratory/TDL) via courier.

Moxifloxacin plasma concentrations were quantified using validated LC–MS/MS methods. Moxifloxacin was extracted from 100 μL of human plasma by precipitation with acetonitrile. Norfloxacin solution (0.1 mL, 10 μg/mL) was used as an internal standard, and was added to calibrators, quality control or thawed plasma samples (100 μL aliquot) in a 2 mL polypropylene tube. 0.5 mL acetonitrile was added to the tube and the samples were mixed for at least 2 min, and then centrifuged at approximately 13,000 rpm for at least 2 min. 100 μL of supernatant was diluted with 1 mL of assay diluent (de-ionised water, formic acid, triethylamine 1000:1:1) in a 2 mL autosampler vial and submitted to analysis by LC–MS/MS on an Applied Biosystems API 4000 LC–MS/MS machine, a 100 mm × 3.0 mm Onyx Monolithic C18 column and Agilent 1290 multi-sampler and pump.

Samples below the lower limit of quantification were set to zero for statistical purposes. Samples below the lower limit of quantification in the terminal phase (after the last quantifiable concentration) were omitted from the analysis.

### Mealtimes

On each study day, no breakfast was provided, the lunch was given at 6 h post start of clamp and the dinner 10 h post.

### Statistical analysis

The primary analysis was based on a concentration effect model describing the placebo corrected change from average baseline as a function of corresponding change in glucose concentration. Sex was incorporated into the model, to explore possible sex differences.

The effect on QTcF was calculated using the concentration effect analysis based on the placebo-corrected change from average baseline for both QTcF and glucose concentrations (giving ΔΔQTcF and ΔΔGlucose). A linear effects model was used and its appropriateness confirmed. A linear mixed effects model was fitted relating ΔΔQTcF to ΔΔGlucose. The model used sequence as fixed factor (study day) and ΔΔGlucose, moxifloxacin concentration and sex as fixed covariates. Where possible, the interaction ΔΔGlucose *Moxifloxacin was added to the model. Random effects for intercept and ΔΔGlucose were assumed, but no fixed intercept was used. Both the day of effect and the ΔΔGlucose * Moxifloxacin effect were used to evaluate the potential further impact of moxifloxacin on QTcF when administered in addition to glucose on day 3. The predicted effect on QTcF for various increases in glucose level was presented together with two-sided 90% CIs, by sex and overall. Specifically, the effect on QTcF was evaluated at the peak glucose concentration (estimated around the 2H time point) when glucose was given alone, and just after the C_max_ for moxifloxacin at 1.75H.

Similar models were fitted to explore the effects of glucose on JTp_c_J and on TpeakTend (TpTe). Exploratory models were also investigated, adding ΔΔPotassium as a covariate and the insulin regimen as a fixed factor in the primary analysis model.

## Results

### Hyperglycaemia prolonged QTcF

Volunteers displayed a statistically significant increase in the placebo corrected duration of QTcF in correlation with hyperglycaemia with a peak mean increase of 13 ms at 2 h (Fig. 2). The observed effect was statistically significant at all timepoints. The prolongation of QTcF occurred early in glucose administration and plateaued from 1 h while serum glucose concentration was still rising.

Male and female volunteers both displayed QTcF-prolongation (Fig. 3) during hyperglycaemia but the peak mean increase in QTcF prolongation in female volunteers was longer (16 ms) than in males (10 ms).

### Prolongation of the QTcF during hyperglycaemia was primarily driven by JTpcJ prolongation

**Fig. 4:**
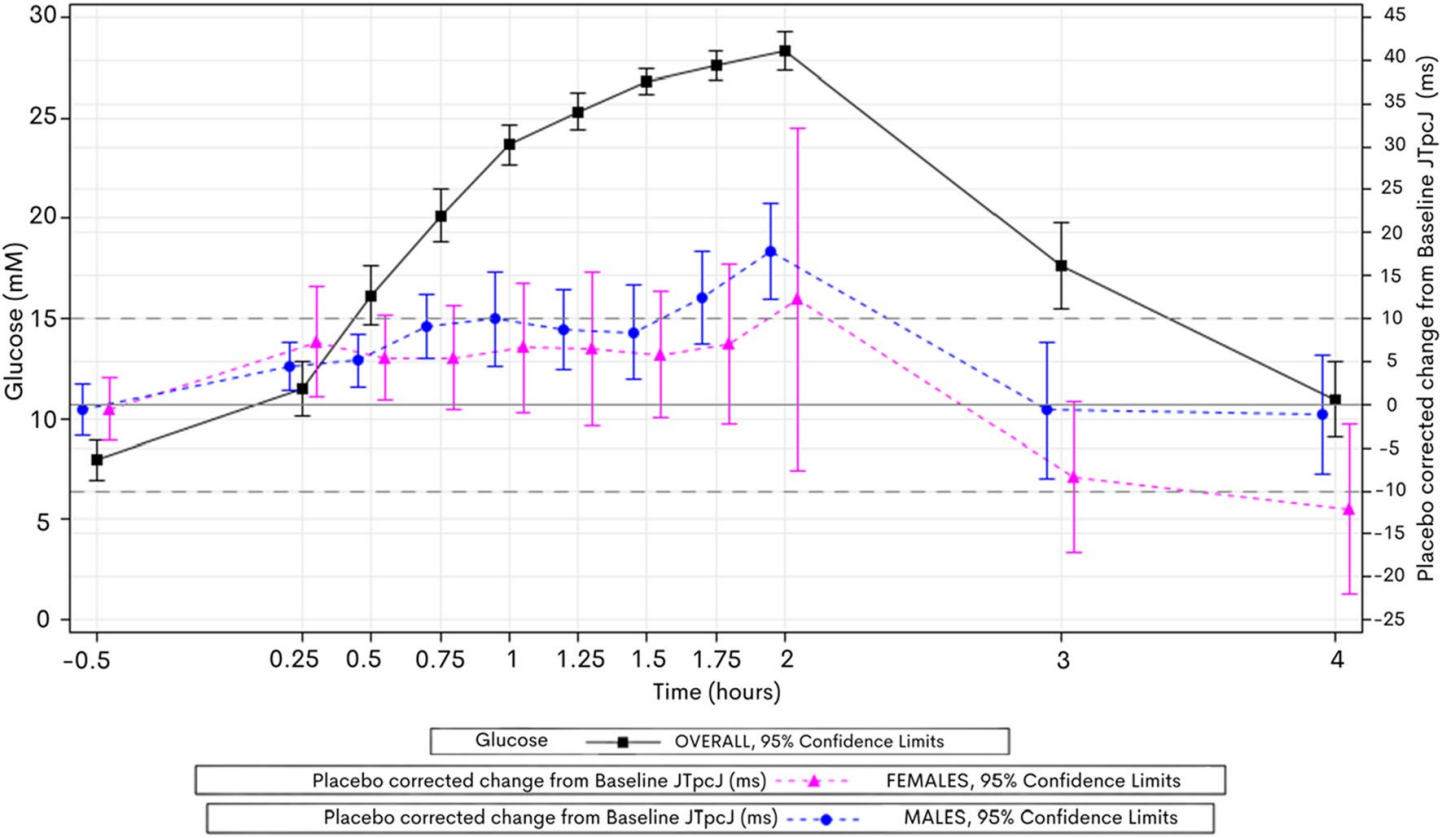
Mean ∆∆JTp_c_J over time vs Mean Glucose over time on day 1—by gender—subinterval analysis of J-Tpeak (corrected) revealed a mean placebo corrected increase of 18 ms in men and 12 ms in female volunteers.

On subinterval analysis, all volunteers showed a prolongation in heart-rate corrected J-Tpeak_c_ (Johannesen) (JTp_c_J) with a peak mean increase of 15 ms at 2 h, which again shortened to below baseline after glucose administration was stopped and insulin given. The peak at 2 h of mean JTp_c_J prolongation was less in female volunteers (12 ms) and greater in males (18 ms) (Fig. 4).

### Men displayed TpTe-shortening in response to hyperglycaemia while women did not

In the gender subgroups, males displayed statistically significant nadir TpTe-shortening below baseline at 2 h of -7 ms (Fig. 4—Supplementary material), offsetting their greater prolongation effect on JTp_c_J, and therefore reducing QTcF prolongation overall. In the female subgroup, there was no significant effect on TpTe versus baseline.

### Insulin rapidly corrects QTcF prolongation

After insulin was initiated at the 2 h timepoint, the QTcF rapidly shortened to below baseline levels by around -10 ms by hour 4, despite glucose remaining above baseline levels.

### Moxifloxacin induced QTc prolongation was additive in hyperglycaemia

**Fig. 5:**
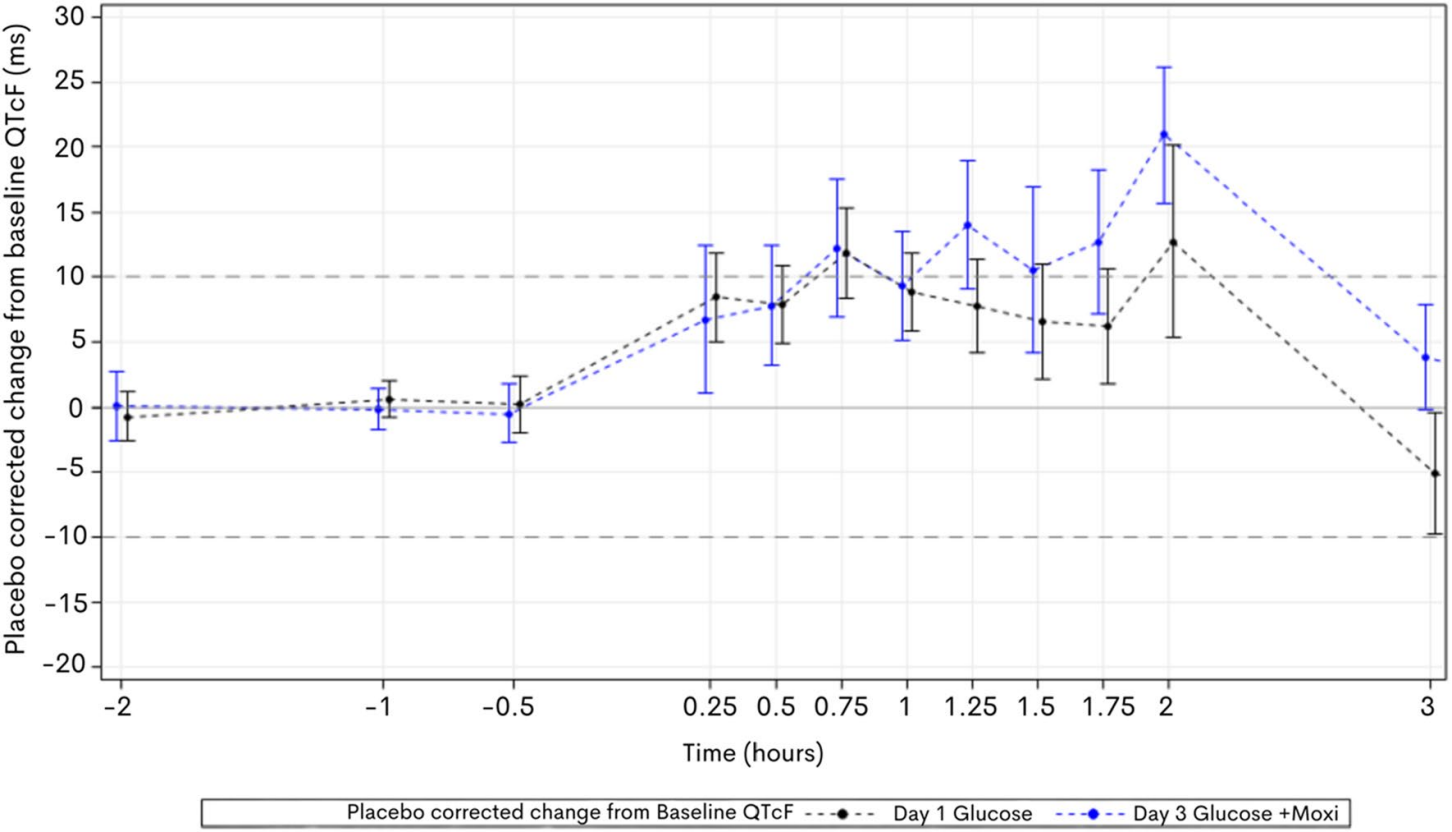
Mean ∆∆QTcF over time vs Mean Glucose concentration over time, on day 1 (glucose plus placebo—black) and day 3 (glucose administration with intravenous moxifloxacin—blue). On day 3, volunteers were administered hyperglycaemic clamp and 300 mg of IV moxifloxacin over 45 min and QTcF compared to placebo on day 2 to derive the placebo corrected change. Day 3 peak mean placebo corrected increase in QTcF was 20 ms at peak glucose concentration (2 h) (blue line) compared to the day 1 peak increase of 13 ms with glucose alone (black line).

When moxifloxacin was co-administered in the hyperglycaemic state, volunteers displayed additional QTcF-prolongation. Peak mean placebo corrected QTcF-prolongation at 2 h with moxifloxacin administration in combination with glucose was 22 ms, compared to 13 ms at the same timepoint with glucose alone (Fig. 5).

The effect of moxifloxacin administration in hyperglycaemia on QTcF was the same in both female and male cohorts (peak mean placebo corrected QTcF-prolongation 22 ms at 2 h) compared to glucose alone (15 ms and 10 ms, respectively).

The additional mean QTcF prolongation suggests that 1 µg/mL plasma moxifloxacin yields an increase in the QTc interval of 3.04 ms, which is in keeping with previously published studies of the concentration effect of moxifloxacin on QTc interval [5, 30, 39]

### Prolongation of the JTp_c_J interval appears to drive moxifloxacin induced QTc prolongation in hyperglycaemia

On subinterval analysis, the additional effect of moxifloxacin in hyperglycaemia was predominantly driven by increased prolongation of JTp_c_J with a similar peak placebo corrected increase at 2 h of 24 ms in females and 23 ms in males versus 12 ms and 17 ms in hyperglycaemia alone, respectively.

TpTe was again significantly shortened in the male cohort (Fig. 5—Supplementary material), but this effect appeared to be diminished in the presence of moxifloxacin, nearing baseline at 2 h, and contributing to a more prolonged QTcF overall compared to hyperglycaemia alone.

### Moxifloxacin PK results

On day 3, Moxifloxacin concentrations increased in line with previously published PK studies, reaching a geometric mean C-max of 2.1 ug/mL at 1.75 h. (Fig. 6—Supplementary material).

### Model-based concentration effect analysis

Modelling was then used to estimate the concentration effects of glucose in isolation, and the combined effect of glucose and moxifloxacin on cardiac subinterval duration. The parameters for the primary model are shown in Table 2—Supplementary material.

The primary linear model showed a highly statistically significant influence of glucose on placebo corrected change from baseline QTcF (*F* Value 67.51, *p* =  < 0.0001). Moxifloxacin administration was borderline significant (*F* value 2.18, *p* = 0.14) but with limited effect, and the interaction between glucose and moxifloxacin was also borderline significant (*p* = 0.067) suggesting the effect of moxifloxacin in addition to glucose on the QTcF is additive, not exponential, reflected in the similar regression line slopes.

Concentration-effect modelling (not shown) for JTp_c_J showed again a highly significant influence of glucose concentration on JTp_c_J prolongation (*F* 54.59, *p* =  < 0.0001) while the interaction of glucose and moxifloxacin neared but not quite reached significance (*p* = 0.11).

No statistically significant factor influenced overall TpTe modelling in the overall cohort.

### Insulin regimen influences QTcF prolongation due to glucose and moxifloxacin

63.4% female subjects used short-acting insulin pump preparations only, which were suspended for the clamp period, while 100% of male subjects used a basal-bolus regimen and took their basal insulin on each study day. Time-course analysis of QTcF by insulin regimen suggested that males (on additional basal long-acting insulin) had a less pronounced QTcF prolongation at 2 h during hyperglycaemic clamp compared to no additional insulin (most females). Whilst the level of hyperglycaemia was maintained at the same level of 25 mM in all volunteers, more glucose had to be given to those using long-acting insulins (Table 3—Supplementary material).

When added as a covariate to the primary model, the type of insulin regime was found to have an influence on the duration of QTcF (Table 4), although this did not quite reach significance (*F* 2.35, *p* = 0.12). When accounting for the insulin regimen, the interaction between glucose and moxifloxacin did reach statistical significance (*F* 3.75, *p* = 0.05).

## Discussion

### The cardiac action potential and QT interval

The QT interval represents the cumulative surface depolarization and repolarization of the myocardium and varies with heart rate. Various methods to correct for heart rate (QTc) have been reported in the literature including Bazett, Fridericia, Framingham and Hodges, with Fridericia shown to be more accurate and closer associated with 30-day mortality [41]. Individual heart rate corrections have been suggested to better reflect the QT/RR relationship with higher heart rates [11]. QTc prolongation is a risk factor for ventricular arrhythmia, particularly Torsades de Pointes (TdP) and associates strongly with mortality in a wide range of conditions, including diabetes mellitus [8]. The QT interval has been further characterised into the subintervals J-Tpeak and Tpeak-Tend, the latter of which has been shown to be independently predictive of ventricular arrhythmia and death in acute ST-elevated myocardial infarct [29]. Automated methods for heart-rate correction of J-Tpeak (J-Tpeak_c_) have been reported [19].

QTc prolongation occurs in between 17 and 44% [26, 36] of type I and II diabetes mellitus, although the underlying mechanisms remain unclear. It is likely in a multi-system disease such as DM it is a combination of many the factors described in the literature [Fig. 1 – Supplementary material] together that cumulatively prolongs QTc. Roden [32] et al. described this as a ‘repolarization reserve’ that needs to be overcome before pathological prolongation leading to ventricular arrhythmia occurs.

Factors of relevance described in the literature in DM include advanced glycated end-product deposition in the myocardium [3], myofibroblast ‘switching’ [14], autonomic neuropathy [12], inflammation via TNF-alpha and IL-1b [43], renin–angiotensin system activation [7], and blood glucose levels. [Fig. 1- Supplementary material].

### Hyperglycaemia causes QTc prolongation

Here, we demonstrated a consistent significant prolongation of the QTcF in hyperglycaemia, our extended analysis showed a 0.54 ms QTc prolongation per mM increase of glucose. This compares similarly to other metabolites, for each mM increase the effect on the QTc has been reported [27] as follows: Calcium −22.3 ms, Magnesium + 6.4 ms, Potassium −2.8 ms. Glucose has a wider physiological range, meaning total impact is similar in significant deviations from the norm. In comparison to recognised QTc-prolonging medication, dofelitide [10] has been shown to prolong QT by a similar amount 7–16 ms at a dose of 0.75 mg. Sevoflurane anaesthesia prolonged QTc by around 46 ms[20].

While hypoglycaemia is well recognised in clinical practice as a QTc-prolonging factor, an increasing body of evidence, including the results presented here, show that hyperglycaemia also causes consistent QTc prolongation.

Zhang et al. [44] demonstrated this dual effect in impairment of *hERG* potassium channels (IK_r_) in vitro, proposing a depletion of *ATP* in hypoglycaemia impairs IK_r_ function and an overproduction of reactive oxygen species (ROS) in hyperglycaemia impairs function of the same ion channel but by a different mechanism. Diabetic animal models show a similar pattern of chronic downregulation of *hERG* protein, particularly in rabbit models [45], where downregulation was partially normalised by insulin administration, independent of blood glucose. The authors theorised that insulins’ antioxidant properties might also be beneficial. The *hERG* protein is also expressed in the beta-cells of the pancreas, where downregulation in Long QT Syndrome 2 (KCNH2) [18] patients increases insulin release in response to raised blood glucose, suggesting the relationship between insulin, glucose and *hERG* expression in the myocardium is part of a multi-organ axis.

Gordin et al. [15] conducted a similar study to ours under controlled conditions in 22 type 1 diabetes patients and 13 non-diabetic controls. Importantly, all type 1 diabetes patients were continued on regular glargine, while non-diabetic controls were given somatostatin to suppress endogenous insulin—in effect creating a group of insulin-deficient patients with exogenous insulin, and a group of insulin-replete volunteers without any endogenous insulin. Hyperglycaemia induced QTc prolongation in both groups, but this effect was greater in the non-diabetic controls than patients with type 1 diabetes (peak QTcF + 44 ms versus peak QTcF + 28 ms). QTc prolongation in this study peaked early (< 60 min) and then plateaued, a finding replicated in our study. The effect of endogenous insulin suppression is unclear; in healthy volunteers Marfella et al. [24] demonstrated the same prolongation of QTc in healthy volunteers exposed to intravenous hyperglycaemic clamp, without any discernible difference when somatostatin was administered.

In real-world studies in patients with type 1 diabetes, the degree of QTc prolongation is suggested to be longer with hyperglycaemia than hypoglycaemia. [21] Stern et al. [35] identified a positive association with resting QTc and HBA1C in adolescents with type 1 diabetes, and an inverse correlation with hypoglycaemic episodes—suggesting chronic hyperglycaemia is the more important risk factor for QT prolongation in this cohort. Charamba et al. [4] studied hyperglycaemia and QT interval changes in real-time, utilising continuous glucose monitoring (CGM) in combination with a 7 day 12-lead ECG monitor in 17 T1DM patients in Ireland. The mean QTc was significantly longer during hyperglycaemia. This study was limited, however, by the small patient population and amount of missing data from the final analysis. Hyperglycaemia associated QTc prolongation is described in the critically ill [31], with raised blood glucose correlating with QTc prolongation and predictive of mortality. However, previous studies have shown that QTc prolongation is also associated with degree of sickness overall (APACHE II) [2] and, therefore, this may be confounding.

Overall, the body of literature highlights the importance of hyperglycaemia-induced QTc prolongation in diabetes mellitus, a finding replicated here.

### Insulin rapidly corrected QTc and shortened below baseline

At 2 h, the cessation of glucose and commencement of insulin rapidly corrected the QTc, significantly shortening below baseline. Insulin is well recognised to act independently on the cardiovascular system, as detailed by Dubo et al. [13], specifically influencing cardiac output, stroke volume, heart rate, mean arterial pressure and systemic vascular resistance. In alloxan-induced diabetic models of dogs Lengyel et al. [22] demonstrated a significant loss of Ito and IK_s_ current amplitudes, an effect which was partially reversed by treatment with insulin with Ito and fully reversed in IK_s_. This effect has also been observed in vitro rat myocytes by Shimoni et al. [34], where incubation of rat myocytes with insulin recovered protein expression of protein Kv4.2, which forms a vital component of Ito_fast_ channels. In knock-out mouse models simulating insulin deficiency in type 1 diabetes, Lopez-Izquierdo et al. [23] demonstrated similar reductions in the Ito_f_ amplitude and prolongation of the QTc. It is possible that insulin’s effect on the QTc may be mediated predominantly by Ito upregulation. This group have previously demonstrated human subjects’ experience J-Tpeak shortening for 4 h following a meal [38], and it may be that this is similarly mediated by the rise in insulin in response to food ingestion.

Although the study set out to observe sex differences the male and female cohorts were unbalanced for their insulin regimen. All male patients were on a basal-bolus regimen and continued to take their regular long-acting insulin through the study period, whereas ~ 60% of the female patients used a subcutaneous pump only and were truly insulin-deplete during the study period. On subinterval analysis we observed a TpTe shortening during hyperglycaemia in men rather than women, and this may be due to the presence of exogenous insulin, but the sample size and unbalanced cohorts limits our analysis.

### J-Tpeak is the predominant underlying subinterval change driving QTc prolongation in hyperglycaemia and additional moxifloxacin

We observed a primary prolongation of QTc in hyperglycaemia driven by J-Tpeak prolongation, an effect which was partially ameliorated by TpTe shortening in men as described above. The additive effect of moxifloxacin was primarily seen in J-Tpeak prolongation, although reduction in the TpTe shortening observed in men in hyperglycaemia also contributed. Moxifloxacin has been demonstrated to block equally both IK_r_ and IK_s_ at on multiple drug panel testing [9]. This additive effect of IK_r_ blockade in both hypo- and hyperglycaemia was explored in extirpated guinea pig hearts by Hreiche et al. [17]. Action potentials were measured with buffer solutions containing 1.5 and 20 mM glucose combined with selective IK_r_ blockade (Dofetilide) and selective IK_s_ blockade (chromanol 293B). Both hypo- and hyperglycaemia prolonged the myocyte action potential, but the greater effect was seen in hyperglycaemia. Both hyper and hypoglycaemia potentiated dofetilide-induced action potential prolongation from an additional 17 ms to 24.2 ms. The presence of hypo- and hyperglycaemia made no difference to IK_s_ blockade, which was overall less impactful (around 7.5 additional ms), an effect size similar to that seen with hyperglycaemia alone. When both IK_s_ and IK_r_ were blocked simultaneously, the effect size was additive (around 35 ms), but there was no variation seen with hypo- or hyperglycaemia as with dofetilide alone. Conversely, in rabbit myocytes Hegyi et al. [16] demonstrated no change in action potential in hyperglycaemia alone, but selective IK_s_ blockade combined with high concentrations (30 mmol) of glucose did cause QTc prolongation. IK_r_ inhibition-induced QTc prolongation was again significantly longer in hyperglycaemic versus euglycaemic states, as was Ito inhibition. This highlights the potential role of multiple voltage-gated ion channels in hyperglycaemia-induced QTc prolongation and emphasises the ‘multiple hit’ hypothesis we have explored here; the combination of drug-induced QTc prolongation and hyperglycaemia.

This effect has been observed in real-world studies; sevoflurane-induced Torsades de Pointes occurred in a patient with poorly controlled type II diabetes and hyperglycaemia at the time of the event [40]. In a further study of sevoflurane-induced QTc prolongation [20], chronic hyperglycaemia (HBA1c > 6.5) was a significant risk factor for QTc prolongation, although Tpeak-Tend_c_ was unchanged.

### Sex differences in QTc risk

This study recorded sex differences in responses to QTcF with hyperglycaemia and moxifloxacin, particularly in subinterval changes. In healthy men and women, differences in their electrophysiology are well recognised with women exhibiting longer QTc intervals and being at greater risk of developing TdP. Because women have a longer constitutive QT interval, the increase in QTcF duration in response to hyperglycaemia may present a risk for development of TdP and SCD, complementary to the observation that diabetic women are more at risk of SCD than their male counterparts in long QT syndrome [28].

Sex differences were further complicated by the incidental new finding of a possible congenital long QT syndrome patient ('intermediate’ based on Schwartz score) in the female cohort, with QTc prolongation > 500 ms in hyperglycaemia. This may explain the wider variability seen in the female cohort versus the male cohort.

While we observed changes in QTc with exogenous insulin regimen and glucose levels, hormones such as glucagon, c-peptide and ghrelin are intrinsically linked to these biochemical pathways, and future studies will examine these hormones in addition.

Future studies will also explore whether the same observations would be made in patients with type II diabetes mellitus, especially those with high insulin resistance. Early stages of type 2 diabetes are marked by hyper-insulinemia, while late stages are insulin-deficient which adds complexity to studying this population.

There are limitations to our study. First, the study is small, and caution must be exercised drawing conclusions applicable to a general diabetic population. However, the results were obtained under highly controlled conditions and the precision of ECG assessments is state of the art. Second, as detailed above, only T1DM have been studied and the fundamental question as to whether insulin resistance will lead to the same effects as insulin deficiency will have to be answered in a separate study. Third, the male and female cohorts were unbalanced in regard to insulin regimen, therefore, this analysis has been presented as exploratory. Given the potential significant influence of exogenous insulin, this finding should be noted and further explored, specifically on the protective effects of insulin on QTcF prolongation for the same value of blood glucose concentration.

## Conclusions

QTc prolongation is a risk factor for SCD and has been known to occur in the presence of hyperglycaemia, as demonstrated emphatically here. QTc-prolonging drugs such as moxifloxacin further prolong the QTc in an additive way.

Our study suggests that QTc-prolonging drugs should be administered to diabetic patients with caution, particularly if their glucose control is poor. A diabetic patient requiring treatment with a QTc-prolonging drug may be protected from pro-arrhythmic risk when prescribed an insulin regimen that provides a long-lasting insulin baseline, but this remains to be explored in future research.

## Supplementary Information

Below is the link to the electronic supplementary material.

Supplementary file1 (DOCX 1042 kb)

## Data Availability

The datasets used during the current study are available from the corresponding author on reasonable request.
