# Supplementary material for: Effect of hyperglycaemia in combination with moxifloxacin on cardiac repolarization in male and female patients with type I diabetes
Source: Clin Res Cardiol. 2022 May 21;111(10):1147–60. doi: 10.1007/s00392-022-02037-8 (PMC9525410; doi:10.1007/s00392-022-02037-8)
Supplement: Supplementary file 1 — Supplementary file1 (DOCX 1042 kb) [file 392_2022_2037_MOESM1_ESM.docx]

**
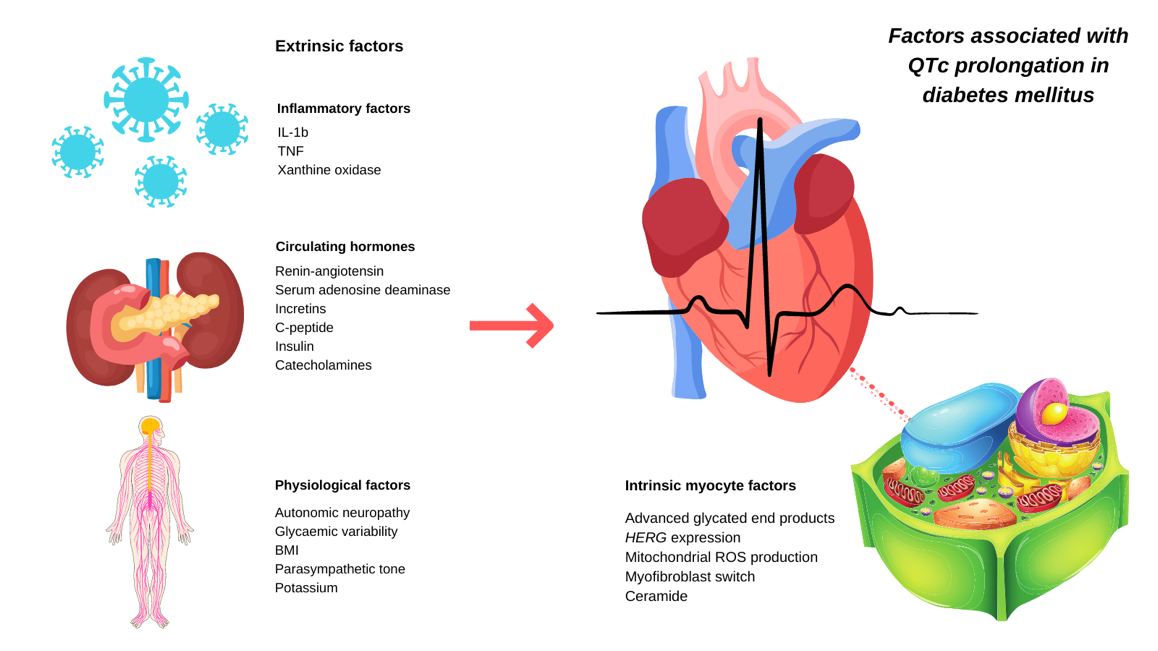
Figure 1**

**Figure 2: Factors implicated in QTc prolongation in human and animal models of diabetes mellitus. (Original diagram. Copyright © 2021 The authors).**

Figure 2:


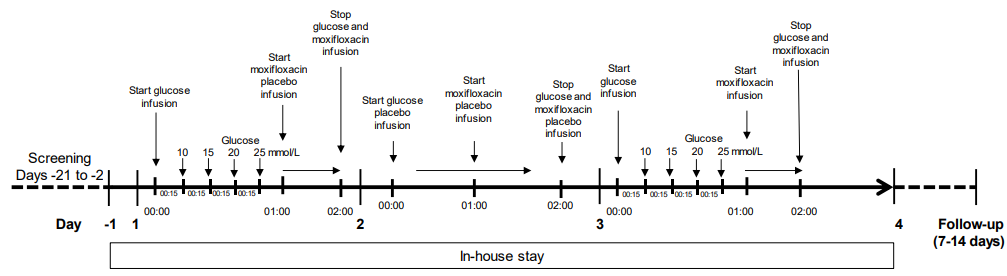


**Figure 3. Study outline**

**Figure 3.**

**
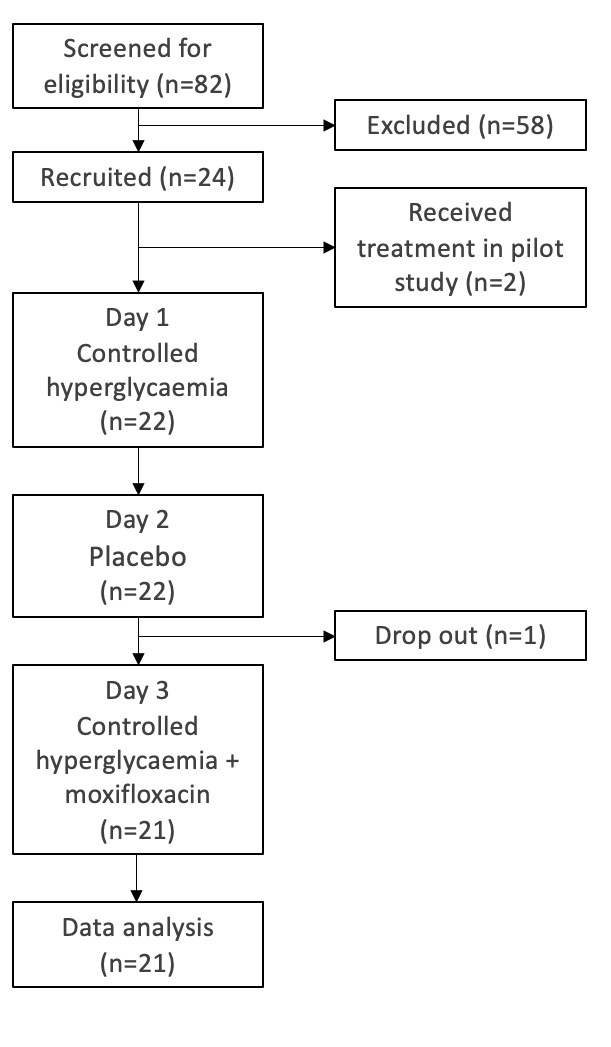
**

**Figure 4. Consort Chart**


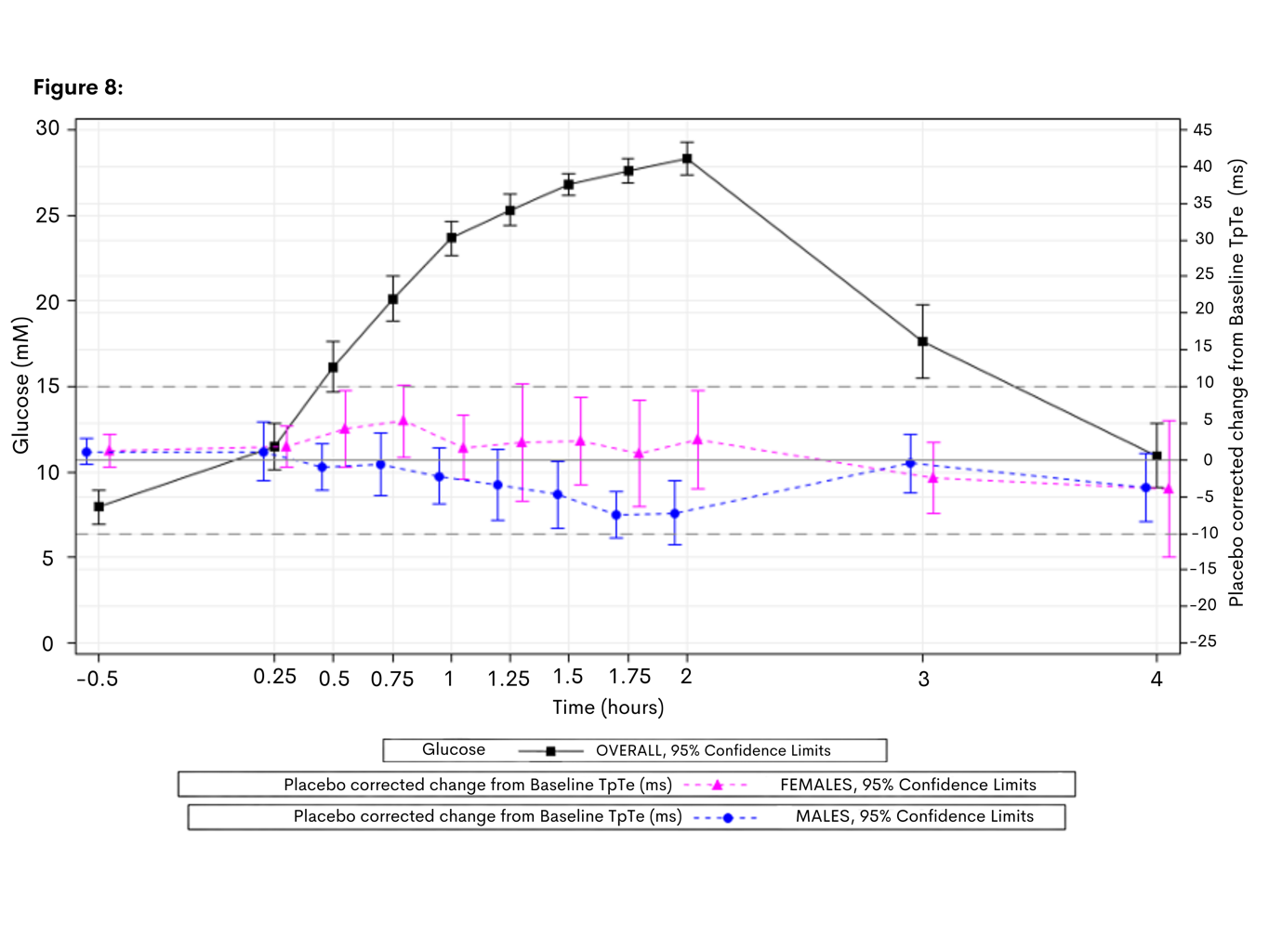


***Figure 4:*** ***Mean ∆∆TpTe over time vs Mean Glucose over time on Day 1 – by gender–*** *subinterval analysis of TpTe revealed a significant shortening of the TpTe subinterval in males, with a nadir of -7 ms (blue) but no significant change in the female cohort.*


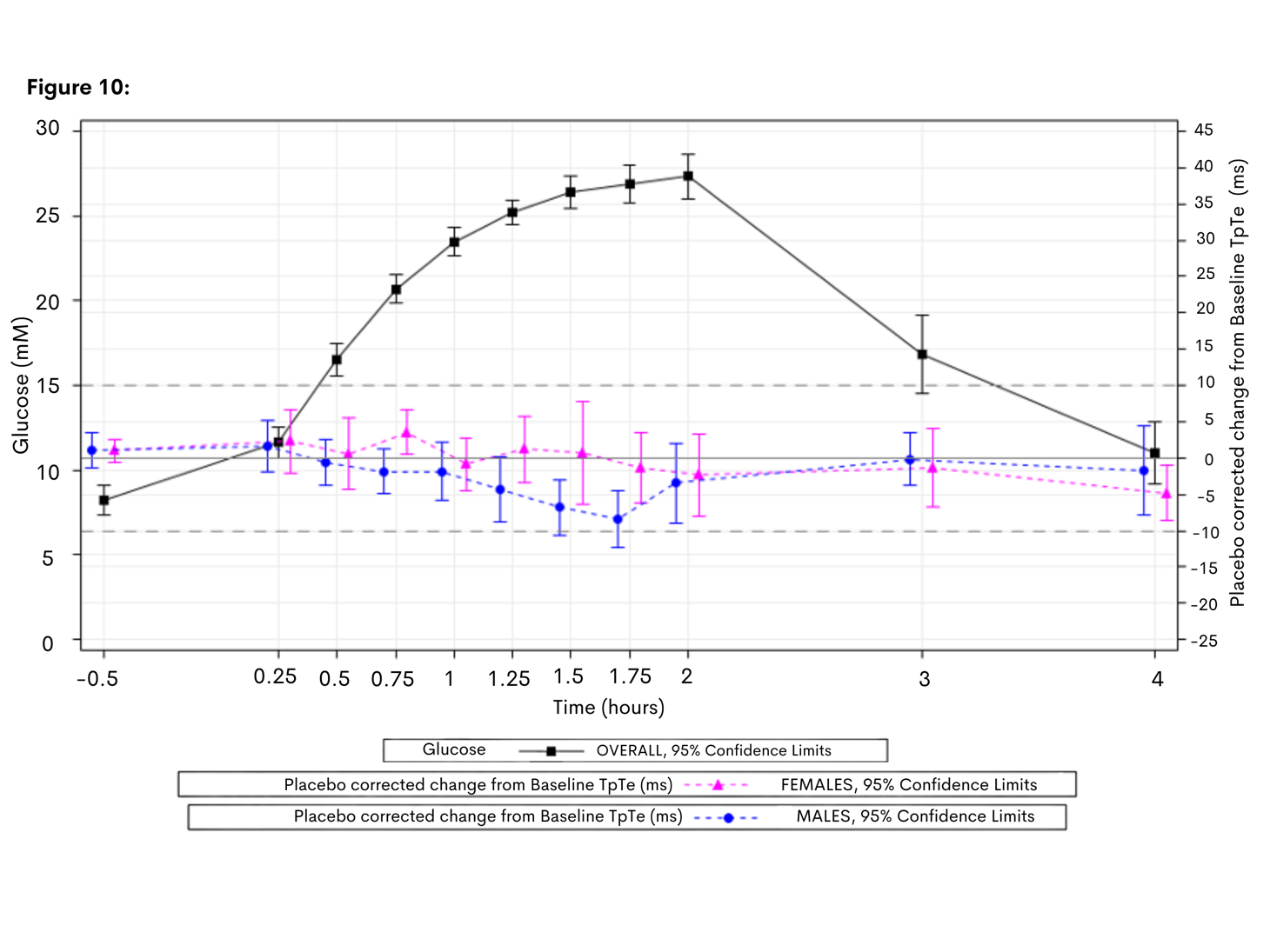


***Figure 5:*** ***Mean ∆∆TpTe over time vs Mean Glucose over time on Day 3 – by gender–*** *subinterval analysis of TpTe on Day 3 revealed a similar shortening in the male cohort which returned to baseline by around 2 hours, contributing to a more prolonged QTcF overall than compared to hyperglycaemia alone. Again, in females there was no significant change.*

*
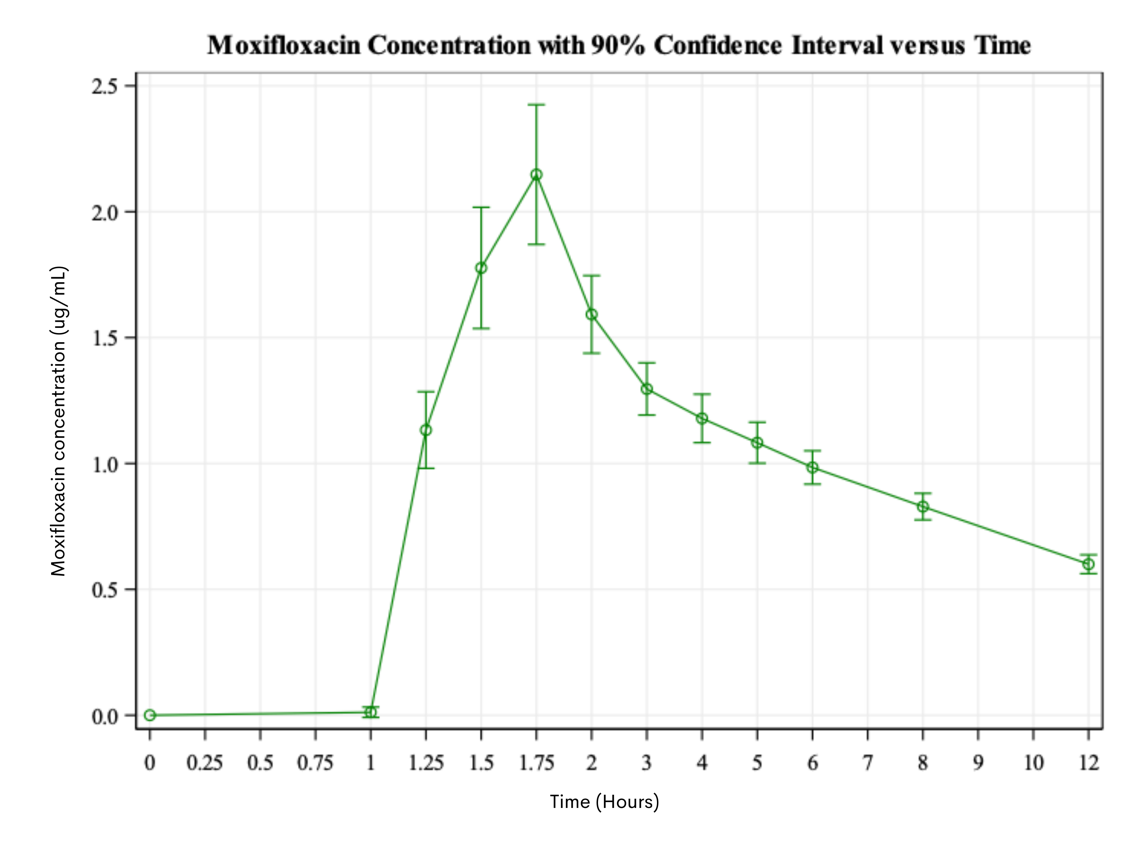
****Figure 6. Moxifloxacin concentration over time on Day 3.*** *C-max was 2.1 ug/mL at 1.75 hours.*

**Tables**

Table 1:

| Demographic Parameter |  | Males | Females |
| --- | --- | --- | --- |
| Sex | n (%) | 10 (47.6) | 11 (52.4) |
| Age [years] | Mean | 25.8 | 27.1 |
|  | SD | 5.07 | 3.39 |
|  | Range | 20-36 | 22-32 |
| Weight [kg] | Mean | 78.1 | 63.6 |
|  | SD | 8.9 | 5.7 |
|  | Range | 64.9 – 93.9 | 54.7 – 73.2 |
| Body Mass Index [kg/m^2^] | Mean | 24.4 | 22.7 |
|  | SD | 2.4 | 1.4 |
|  | Range | 19.7-27.6 | 21.1-26.4 |
| Race, n (%) | Caucasian | 7 (70.0) | 11 (100.0) |
|  | Other | 3 (30.0) | 0 (0.0) |

**Table 1. Cohort Demographics**

| **Type 3 Tests of Fixed Effects** | | | | |
| --- | --- | --- | --- | --- |
| **Effect** | **Num DF** | **Den DF** | **F Value** | **Pr > F** |
| **Study Day** | 1 | 175 | 5.66 | 0.0184 |
| **Sex** | 1 | 19.5 | 3.60 | 0.0728 |
| **Sex* Study Day** | 1 | 176 | 0.06 | 0.8025 |
| **ddGlucose** | 1 | 182 | 67.51 | <.0001 |
| **Moxi** | 1 | 178 | 2.18 | 0.1413 |
| **ddGlucose *Moxi** | 1 | 178 | 3.40 | 0.0668 |

***Table 2: Parameters of the primary model –*** *ddGlucose was a highly significant factor on QTcF duration (p<0.0001) while the interaction between glucose and moxifloxacin (ddGlucose*Moxi) was borderline significant (p = 0.0668) as was sex (p = 0.07)*

|  | | **Day 1** | | | **Day 3** | | |
| --- | --- | --- | --- | --- | --- | --- | --- |
| **Females** |  | Oral (g) | I.V. (g) | **TOTAL (g)** | Oral (g) | I.V. (g) | **TOTAL (g)** |
| N=12 | Mean | 84.7 | 23 | **107.7** | 80.3 | 20 | **100.3** |
|  | SD | 14.1 | 11.7 | **17.9** | 16.7 | 9.4 | **18.3** |
| **Males** |  | Oral (g) | I.V. (g) | **TOTAL (g)** | Oral (g) | I.V. (g) | **TOTAL (g)** |
| N=10 | Mean | 135.2 | 32.9 | **168.1** | 128.7 | 32.9 | **161.6** |
|  | SD | 24.2 | 12.5 | **22.9** | 29.9 | 9.7 | **34** |

***Table 3: Total glucose administration to male and female cohorts.*** *The mean total glucose requirement to maintain levels of 25 mM blood serum glucose for males was 56% greater on Day 1 and 61.1% on Day 3 versus females.*

| **Type 3 Tests of Fixed Effects** | | | | |
| --- | --- | --- | --- | --- |
| **Effect** | **Num DF** | **Den DF** | **F Value** | **Pr > F** |
| **ddGlucose** | 1 | 183 | 66.89 | <.0001 |
| **Moxi** | 1 | 177 | 2.39 | 0.1236 |
| **ddGlucose * Moxi** | 1 | 176 | 3.78 | 0.0534 |
| **Study Day** | 1 | 174 | 5.64 | 0.0186 |
| **Sex** | 1 | 17.1 | 0.06 | 0.8055 |
| **Sex* Study Day** | 1 | 175 | 0.06 | 0.8028 |
| **Insulin Regimen** | 2 | 18.3 | 2.35 | 0.1236 |

**Table 4: Insulin regime as an additional covariate to the primary linear model –** when insulin regimen was added as a covariate to the primary linear model the ddGlucose remained highly significant, and insulin regimen itself reached neared statistical significance (p=0.12). Interestingly once insulin regimen was accounted for ddGlucose * Moxi reached significance (F 3.78 p=0.05).
